# Objective assessment of diagnostic image quality in CT scans: what radiologists and researchers need to know

**DOI:** 10.1186/s13244-025-02037-y

**Published:** 2025-07-10

**Authors:** Eva J. I. Hoeijmakers, Bibi Martens, Joachim E. Wildberger, Thomas G. Flohr, Cécile R. L. P. N. Jeukens

**Affiliations:** 1 Department of Radiology and Nuclear Medicine, Maastricht University Medical Centre+, Maastricht, The Netherlands; 2 CARIM School for Cardiovascular Diseases, Maastricht University, Maastricht, The Netherlands; 3 GROW School for Oncology and Reproduction, Maastricht University, Maastricht, The Netherlands

**Keywords:** Tomography (X-ray computed), Diagnostic imaging, Image quality, Automation

## Abstract

**Objectives:**

Quantifying diagnostic image quality (IQ) is not straightforward but essential for optimizing the balance between IQ and radiation dose, and for ensuring consistent high-quality images in CT imaging. This review provides a comprehensive overview of advanced objective reference-free IQ assessment methods for CT scans, beyond standard approaches.

**Methods:**

A literature search was performed in PubMed and Web of Science up to June 2024 to identify studies using advanced objective image quality methods on clinical CT scans. Only reference-free methods, which do not require a predefined reference image, were included. Traditional methods relying on the standard deviation of the Hounsfield units, the signal-to-noise ratio or contrast-to-noise ratio, all within a manually selected region-of-interest, were excluded. Eligible results were categorized by IQ metric (i.e., noise, contrast, spatial resolution and other) and assessment method (manual, automated, and artificial intelligence (AI)-based).

**Results:**

Thirty-five studies were included that proposed or employed reference-free IQ methods, identifying 12 noise assessment methods, 4 contrast assessment methods, 14 spatial resolution assessment methods and 7 others, based on manual, automated or AI-based approaches.

**Conclusion:**

This review emphasizes the transition from manual to fully automated approaches for IQ assessment, including the potential of AI-based methods, and it provides a reference tool for researchers and radiologists who need to make a well-considered choice in how to evaluate IQ in CT imaging.

**Critical relevance statement:**

This review examines the challenge of quantifying diagnostic CT image quality, essential for optimization studies and ensuring consistent high-quality images, by providing an overview of objective reference-free diagnostic image quality assessment methods beyond standard methods.

**Key Points:**

- Quantifying diagnostic CT image quality remains a key challenge.
- This review summarizes objective diagnostic image quality assessment techniques beyond standard metrics.
- A decision tree is provided to help select optimal image quality assessment techniques.

**Graphical Abstract:**

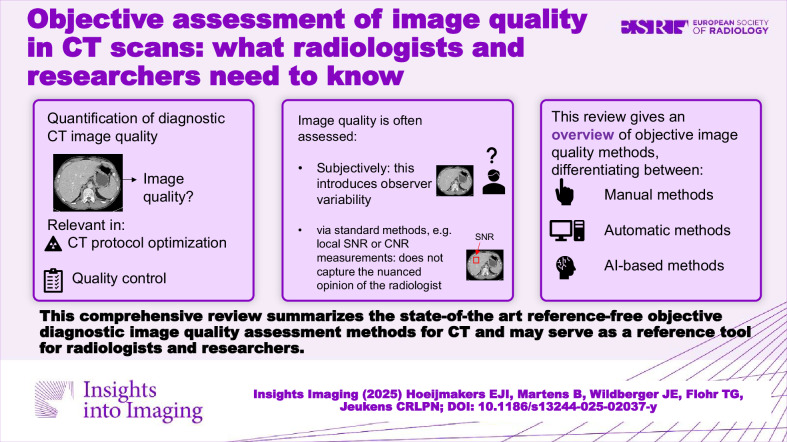

## Background

Computed tomography (CT) has revolutionized conventional X-ray-based medical imaging by enabling 3D visualization of internal structures. However, this advancement comes with an increase in radiation exposure, which raises potential health risks to patients. A key challenge in CT imaging remains in concurrently optimizing radiation dose and diagnostic image quality (IQ) [1]. While there are well-defined metrics for straightforward quantification of radiation dose, accurate quantification of diagnostic IQ is far more complex and requires a comprehensive understanding of how various IQ metrics (e.g., noise, spatial resolution, contrast) influence IQ and how to evaluate them effectively [2–4].

Diagnostic IQ can be evaluated subjectively and objectively. Subjective methods, such as radiologist assessments using a Likert scale, remain to this day the gold standard. While this method is widely used, it has several drawbacks, such as high inter- and intraobserver variability, reader fatigue and a tendency to avoid extreme ratings [5–7]. To address some of these issues, a new subjective assessment method using pairwise comparisons has been proposed, which shows improved consistency between observers [8]. However, this approach cannot completely eliminate observer variability.

To objectively evaluate diagnostic IQ of CT scans, various standard physics-based techniques, such as region-of-interest (ROI)-based noise measurements, contrast-to-noise ratio (CNR), and signal-to-noise ratio (SNR) calculations, have traditionally been employed [2, 9]. While these techniques provide quantitative insights into certain aspects of IQ, they often lack the ability to capture the nuanced perceptual preferences of radiologists.

Objective assessment methods are generally categorized into two approaches: reference-free IQ assessment and full-reference IQ assessment [10]. Full-reference methods compare IQ to a predefined reference image. However, obtaining a reference image for every scenario is challenging and might even be impossible due to variability in patient anatomy, pathology, and imaging. In contrast, reference-free IQ assessment methods evaluate IQ based solely on intrinsic image properties, such as noise, contrast, and spatial resolution, without the need for a reference.

Ideally, objective IQ assessment methods should be consistent with human observers and allow automated IQ assessment [11]. A fully automated IQ assessment method, implemented directly after CT scan acquisition, would allow for instant feedback if IQ is below a certain threshold. Such a system could also support continuous quality control for CT scanners, ensuring consistently high-quality images and enabling the optimization of the dose-IQ trade-off across large datasets [10, 11].

This review focuses on existing reference-free advanced objective IQ assessment methods, including artificial intelligence (AI) algorithms, beyond the standard objective IQ methods (i.e., ROI-based noise, CNR and SNR analysis). We provide a comprehensive overview based on our own experience in developing, analyzing and applying various IQ assessment methods [8, 12–14]. It is intended to serve as a reference tool for researchers and radiologists when designing studies involving CT scans that require IQ assessment.

## Methods

### Data sources and study selection

For this systematic review, a literature search was performed in PubMed and Web of Science to identify all papers published in peer-reviewed literature until June 2024. The search was conducted using combinations of the terms: *CT, contrast, noise, spatial resolution, image quality, diagnostic, clinical, patient, algorithm, computer-assisted, automated, quantification, model observer, measurement, assessment, machine learning, deep learning, development, proposes, subjective* and *likert*, defined in consensus by all authors.

Studies were included if they met the following criteria:

- Written in English.
- Explicitly discussed, proposed or applied objective quantitative reference-free methods for IQ assessment of CT, including AI-based methods.
- Applied to or applicable to clinical data.

Exclusion criteria were:

- Studies that used only standard quantitative IQ assessment methods, i.e., a ROI-based analysis extracting mean Hounsfield unit (HU) ± standard deviation (SD), SNR, and CNR.
- Studies that lack detailed descriptions of their methods, rendering them non-reproducible.
- Studies focusing solely on artifacts as an IQ measure were also excluded, as artifacts are not directly related to the performance of the scanner self or are only partially influenced by the scanner.
- Studies with full-reference IQ assessment methods (e.g., the Structural Similarity Index Measure).

After removing duplicates, the initial screening was conducted by one author (E.H.) based on the titles to exclude studies that were clearly unrelated to CT imaging. Subsequently, studies that did not meet the inclusion and exclusion criteria were filtered out by reviewing the abstracts and, when necessary, full texts. A second author (C.J.) also reviewed the full texts independently to verify eligibility. In case of disagreement, a consensus was reached through discussion.

Both authors independently performed data extraction from the included studies. The final selection of studies was categorized into four main groups based on the aspect of IQ they addressed: noise, contrast, spatial resolution, and other factors. Within each category, studies were further subcategorized by the type of method used:

- Manual: Techniques that require human involvement for measurement or evaluation, such as manual ROI placement.
- Automatic: Fully deterministic algorithms that operate without human involvement once configured.
- AI-based: (automated) methods using machine or deep learning techniques that automatically learn patterns from data.

Finally, the following information was extracted for each study: the IQ assessment methodology, the body region for which the method is applicable, the key principle, whether the method is ROI-based, key advantages and disadvantages of the method and if and how the method was validated. Based on this information a decision tree was constructed as a tool to help deciding on a relevant method corresponding to the research goal.

## Results

**Fig. 1:**
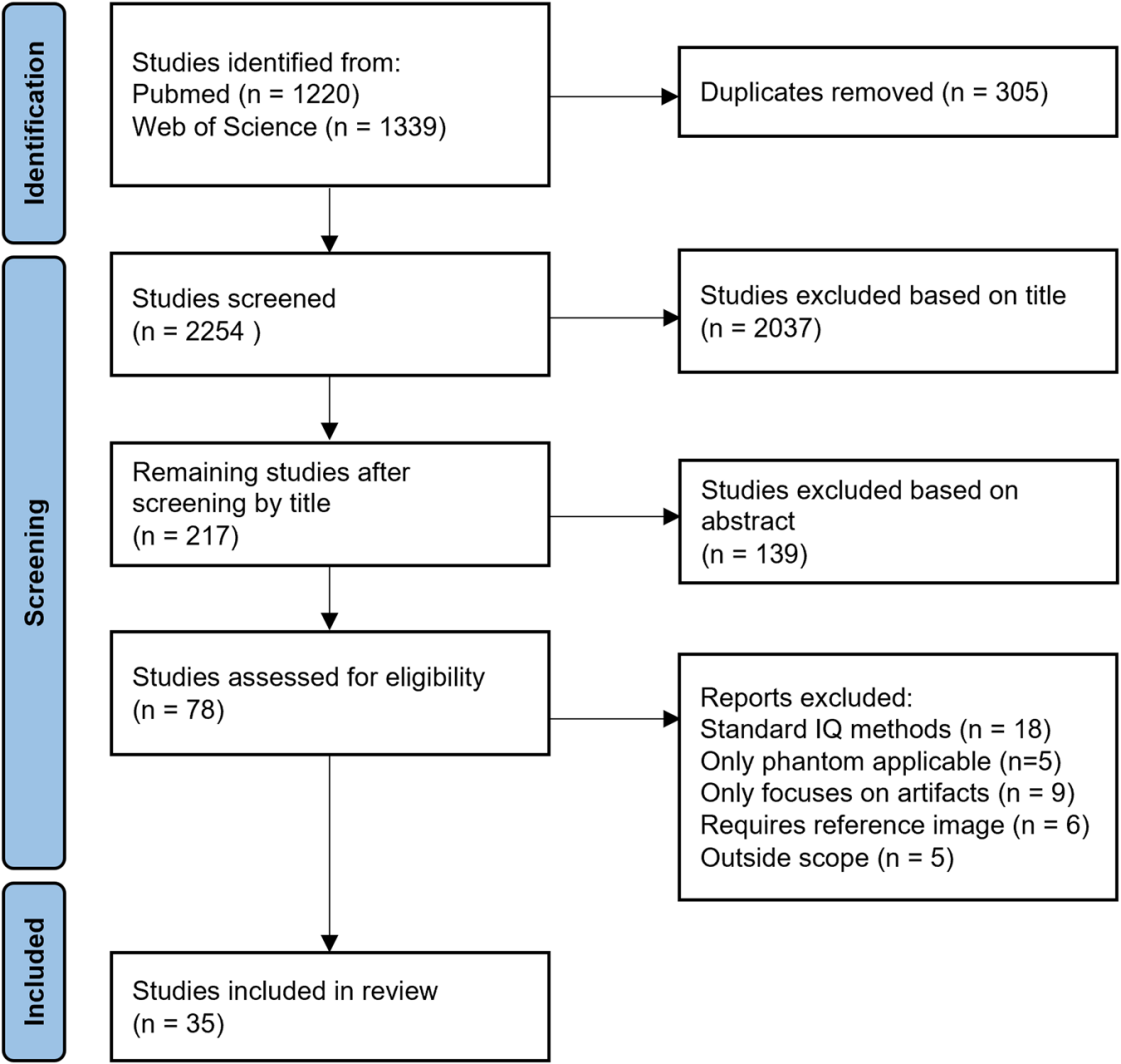
Preferred Reporting Items for Systematic reviews and Meta-Analysis (PRISMA) flowchart of study selection

The Preferred Reporting Items for Systematic reviews and Meta-Analysis (PRISMA) flowchart of the screening process is given in Fig. 1. The primary literature search yielded 2559 studies of which 304 were duplicates, resulting in 2254 studies. From these, 78 studies were selected for full text review, after which 35 studies were included in this systematic review.

**Fig. 2:**
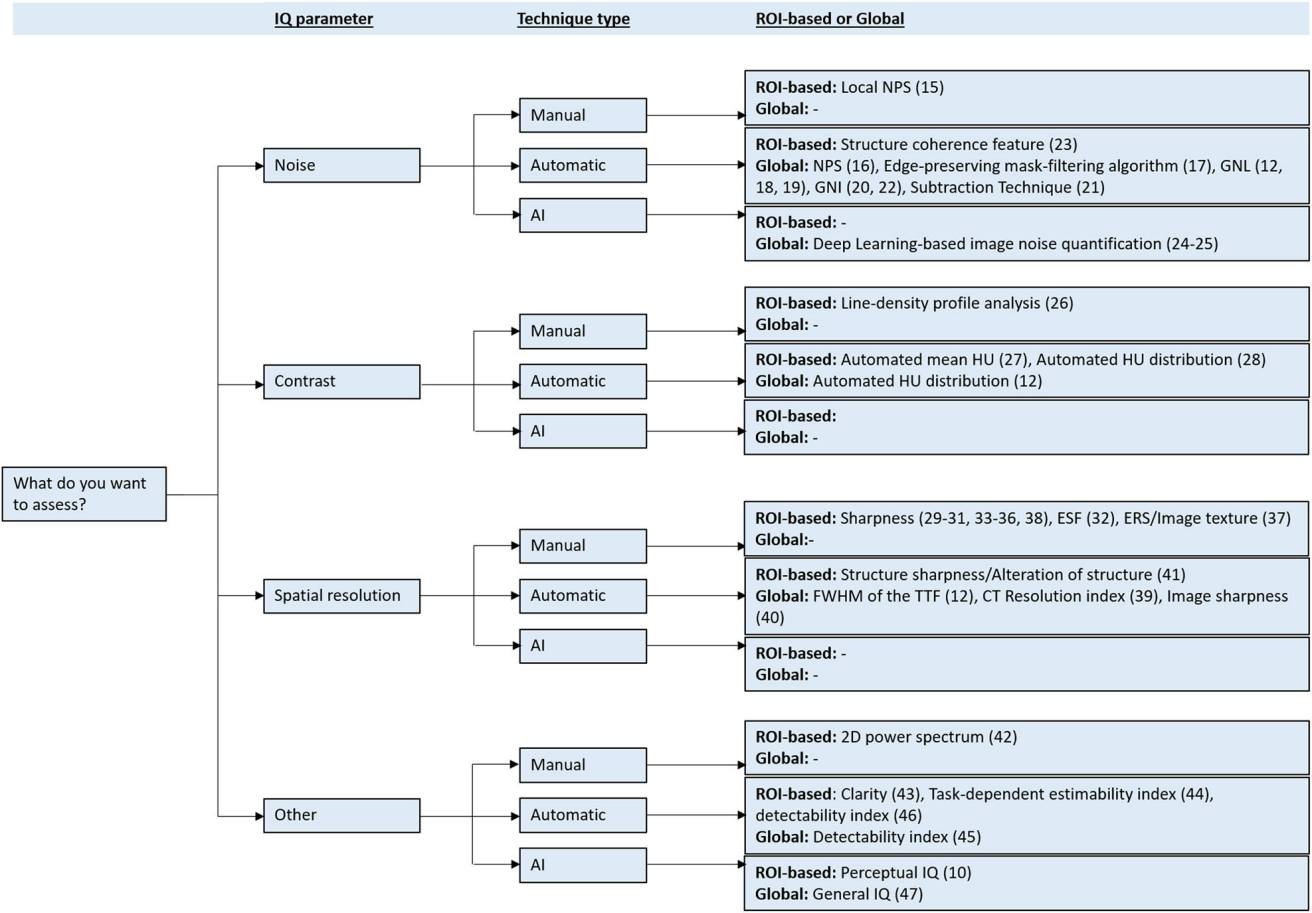
Decision tree providing a structured overview of advanced image quality (IQ) assessment techniques for CT. ROI, region-of-interest; IQ, image quality; AI, artificial intelligence; NPS, noise power spectrum; GNL, global noise level; GNI, global noise index; HU, Hounsfield unit; ESF, edge spread function; ERS, edge rise slope; FWHM, full width half maximum; TTF, task transfer function

The subdivision of the studies in the four categories resulted in: *n* = 12 IQ assessment methods focusing on noise, *n* = 4 on contrast, *n* = 14 on spatial resolution and *n* = 7 on other aspects, where two studies were included in more than one category. Figure 2 gives a decision tree providing a structured overview of the selected objective IQ assessment techniques.

### Noise

Twelve studies reported on noise assessment techniques (Tables 1 and S1 for more details). One study required manual ROI placement [15], nine studies employed automatic noise assessment methods [12, 16–23], and two used AI-based approaches [24, 25].

**Table 1:** Objective CT image quality assessment methods focusing on noise

Study	Methodology	Technique type	Body region	ROI or Global	Key principle
Zhang et al [15]	NPS	Manual	All	ROI	Novel mathematical method that uses the raw count data of PCD-CT scan to obtain a local NPS
Smith et al [16]	NPS	Automatic	Abdomen	Global	Segmenting the liver, selecting a uniform area within it and measuring noise patterns within the data via noise auto covariance
Kortesniemi et al [17]	Edge-preserving mask-filtering algorithm	Automatic	All	Global	Creating a map of filtered local minimum SDs using a moving square mask, forming an IQ score
Christianson et al [18]	GNL	Automatic	Abdomen	Global	Finding the most frequent noise level in areas of homogeneous tissue
Anam et al [19]	GNL	Automatic	All	Global	Finding the minimum standard deviation in any region of the body as a measure for noise
Alsaihati et al [20]	GNI	Automatic	Soft tissue	Global	Enhanced version of Christianson et al [18] but averaging it over slices to obtain the GNI of a complete series
Tian et al [21]	Subtraction technique	Automatic	Abdomen, lung	Global	Subtracting sequential slices and filtering out edges to measure SD in resultant uniform area
Malkus et al [22]	GNI	Automatic	All	Global	Determination of the mode/mean of the SD in air, by subtracting adjacent slices and with an edge-finding algorithm to remove morphological variations
Jeukens et al [12]	GNL	Automatic	All	Global	Combines Malkus et al and Christianson et al by measuring GNL in soft tissue and air
Chun et al [23]	Automated noise measurement with a novel structure coherence feature	Automatic	Regions with fat	ROI	Calculation of the structure coherence feature to determine homogeneous ROIs and using the average SD as noise level
Ketola et al [24]	Deep learning-based image noise quantification framework	AI	Chest	Global	Convolutional neural network trained on anthropomorphic phantom data labeled with true noise maps via 2 consecutive scans
Huber et al [25]	Deep learning-based image noise quantification framework	AI	Chest, head and pelvis	Global	Convolutional neural network trained on anthropomorphic phantom data labeled with true noise maps with per-pixel SDs from 100 replicate scans

*ROI* region-of-interest, *NPS* noise power spectrum, *PCD-CT* photon-counting detector CT, *SD* standard deviation, *GNL* global noise level, *GNI* global noise index, *AI* artificial intelligence

#### Manual methods

Zhang et al reported a manual method determining the local Noise Power Spectrum (NPS) for photon-counting detector CT, which measured noise in localized ROIs using raw detector counts [15]. It eliminates the need for repeated scans (and thus repeated exposure) as is necessary for standard NPS measurements that are applied on phantoms [4]. The method is however not applicable to conventional energy-integrating detector CT data.

#### Automatic methods

Smith et al suggested an automatic method to do NPS measurements in the liver without repeated exposure, assuming the HU variation in the uniform liver as noise after automatic segmentation [16].

Additionally, automatic global noise evaluations were addressed in seven studies [12, 17–22], some of which are related. An early approach by Kortesniemi et al [17] developed an edge-preserving mask-filtering algorithm to create noise maps by calculating the SD in pixel neighborhoods while excluding sharp transitions. This method provides a comprehensive image-wide noise assessment and has the potential for a series-wide application, i.e., including all slices of a series as opposed to a single slice analysis. Subsequently, Christianson et al introduced the Global Noise Level (GNL) method, based on segmentation of tissue types using threshold values, and using the mode of the histogram of pixel-wise SDs as a robust measure of noise [18] applicable in abdominal CT scans. Anam et al extended the GNL to be able to use it anatomy independent by using a threshold-based segmentation of the whole body and selecting homogeneous regions for noise evaluation via the histogram [19]. Further refinement of the GNL was executed by Alsaihati et al, averaging it over slices to obtain the global noise index (GNI) [20]. However, both studies rely on successful threshold-based segmentation of tissue types.

An alternative to the GNL approach was presented by Tian and Samei [21] with a method that involves subtracting adjacent CT slices to eliminate anatomical variations, and filtering out edges, with noise being measured in the resulting uniform regions. However, a key limitation of this approach is the requirement for sufficiently large uniform areas for accurate noise measurement. Building on Tian and Samei’s work, Malkus et al [22] enhance the method’s applicability by showing that noise can be measured in air regions. Subsequently, Jeukens et al combined this result with the results from Christianson et al to obtain a GNL measured in air [12]. Their study showed a significant correlation with subjective IQ scores in abdominal CT scans, demonstrating the feasibility of algorithm-determined objective IQ.

Another alternative approach was proposed by Chun et al, who developed a method based on the structure coherence feature [23]. This technique ‘filters out’ small anatomical structures, preventing them from influencing noise measurements, and calculates noise by averaging the SDs within automatically selected ROIs instead of globally. This method has the advantage of reducing the impact of anatomical detail on noise assessment, making it potentially more accurate in complex clinical images.

#### AI methods

The two AI-based approaches for measuring noise in CT images utilized convolutional neural networks (CNNs) trained on anthropomorphic phantom data. These methods are designed to automate the traditionally manual process of standard deviation (SD) measurements for noise by generating pixel-wise noise maps automatically. Ketola et al developed a CNN trained with true noise (standard deviation, SD) maps obtained from two consecutive scans, focusing exclusively on chest CT [24]. In contrast, Huber et al trained their CNN using true noise maps derived from per-pixel SDs calculated across 100 replicate scans, covering chest, head, and pelvis CT [25]. Both studies validated their methods on clinical data.

### Contrast

Four studies reported on contrast assessment methods (Tables 2 and S2 for more details), of which one required manual placement of an ROI [26] and three were fully automatic [12, 27, 28].

**Table 2:** Objective CT image quality assessment methods focusing on contrast

Study	Methodology	Technique type	Body region	ROI or Global	Key principle
Beer et al [26]	Line-density profile analysis	Manual	Abdomen	ROI	Difference between maximum and minimum HU values on the line
Pallenberg et al [27]	Automated mean HU measurement of aortic CTA volumes	Automatic	Aorta	ROI	Classification of insufficient, optimal or excessive contrast based on the mean HU values in ROIs that were automatically positioned in the CTA volume (CTA image slices were automatically localized as well)
Abadi et al [28]	Automated HU distribution measurement	Automatic	Chest (lung, liver, aorta, spine)	ROI	Median of the distribution of HUs by segmentation of organs and automated ROI placement
Jeukens et al [12]	Automated HU distribution measurement	Automatic	Abdomen	Global	Generating a histogram of HUs after removing air and bone with threshold-based segmentation. Various contrast metrics are defined with the histogram analysis

*ROI* region-of-interest, *HU* Hounsfield unit, *CNR* contrast-to-noise ratio, *SNR* signal-to-noise ratio, *IQ* image quality, *CTA* computed tomography angiography

#### Manual methods

One manual measurement method was included that evaluated the contrast with a line-density profile analysis, by considering minimum and maximum HU values within a 10-mm line crossing tumorous and healthy tissue [26]. While such a method is very effective for targeted evaluations, it has similar disadvantages to the standard CNR method: it offers no information on the overall contrast distribution and merely focuses on contrast between predefined regions.

#### Automatic methods

Three studies used automated approaches for contrast assessment. Pallenberg et al automated contrast measurement in aortic CT angiography (CTA) volumes by using a template-matching technique to automatically place ROIs in the aorta [27]. The reliance on reference templates may restrict the method’s applicability across different patient cases and anatomical variations. In contrast, Abadi et al and Jeukens et al developed broader, automated contrast measurement techniques that analyze the entire CT image rather than relying on specific regions [12, 28]. Abadi et al introduced an automatic method to measure the distribution of Hounsfield units (HUs) across various organs in chest CT scans, starting with body contour segmentation followed by automated ROI placement in the lungs, liver, aorta, and spine [28]. The median HU value within each ROI was used as a contrast measure, allowing for application across both contrast-enhanced and non-contrast-enhanced scans. This approach benefits from standardized ROI placement, which provides consistent contrast measurements within predefined anatomical structures. Although this method does not require manual ROI placement, it still only gives a measure for contrast in specific areas. Jeukens et al offered a different approach by basing contrast measurement on HU histogram analysis of the entire scan volume. Following a threshold-based segmentation to remove background air and bone pixels, a histogram of HU values is generated where the peaks correspond to different soft tissues. Contrast metrics are derived from the histogram’s shape and peaks, providing insights into the distribution and prominence of different tissue contrasts.

### Spatial resolution

Fourteen studies reported on methods to determine spatial resolution (Tables 3 and S3 for more details). The majority (*n* = 10) of the studies were manual methods [29–38], and four studies contained automatic methods [12, 39–41].

**Table 3:** Objective CT image quality assessment methods focusing on spatial resolution

Study	Methodology	Technique type	Body region	ROI or Global	Key principle
Korn et al [29]	Sharpness	Manual	Head	ROI	Sharpness was quantified in terms of the maximal slope (change in HU per pixel) across a line profile perpendicular to the skull circumference
Brodoefel et al (2014) [30]	Sharpness	Manual	Head	ROI	Sharpness was quantified in terms of the gradient or maximal slope (change in HU per pixel) across a line profile perpendicular to the skull circumference
Ernst et al [31]	Sharpness	Manual	Head	ROI	Sharpness was quantified in terms of the maximal slope (change in HU per pixel) across a line profile perpendicular to the skull circumference
Wu et al [32]	Edge spread function	Manual	Head	ROI	Spatial resolution was quantified in terms of the width of the edge spread function (ESF) across line profiles perpendicular to the boundary between the lateral ventricle and surrounding brain parenchyma
Altmann et al [33]	Sharpness	Manual	Head/Neck	ROI	Sharpness was quantified in terms of the maximum slope (change in HU per pixel) across line profiles perpendicular to a border between fat and muscle tissue
Mergen et al [34]	Sharpness	Manual	Coronary arteries	ROI	Sharpness was quantified in terms of the maximum slope (change in HU per pixel) across line profiles perpendicular to the left anterior descending artery
Feger et al [35]	Sharpness	Manual	Coronary arteries	ROI	Sharpness was quantified in terms of the difference between 25% and 75% of the maximal HU value and in terms of the maximal slope (change in HU per pixel) across a line profile perpendicular to the edge of a vessel
Heinrich et al [36]	Sharpness	Manual	Coronary arteries	ROI	Sharpness was quantified in terms of the maximum slope (change in HU per pixel) across a line profile perpendicular to the edge of the vessel lumen
Cao et al [37]	Edge rise slope (ERS) / Image texture	Manual / Manual	Abdomen / Abdomen	ROI / ROI	Sharpness was quantified in terms of the ERS (the ratio between the difference between the first peak and last dip in HU values, divided by their distance) across a line profile perpendicular to the edge of a portal vein / Skewness of the CT number histogram on the relatively uniform area of the liver parenchyma
Johnson et al [38]	Sharpness	Manual	Intestines	ROI	Sharpness was quantified in terms of the edge-width across an ROI crossing the psoas-fat edge
Sanders et al [39]	CT resolution index based on the edge spread function	Automatic	Skin	Global	Spatial resolution was quantified by segmentation of the patient’s body and measuring the edge spread function (ESF) across line profiles perpendicular to the air-skin interface
Jeukens et al [12]	FWHM of the task transfer function (TTF)	Automatic	Skin	Global	Enhanced version of the method of Sanders et al [39] by calculating the FWHM from the ESF
Salimova et al [40]	Image sharpness	Automatic	Lower extremities	Global	Sharpness was quantified in terms of the 2D image gradient on a per-slice basis after automated segmentation of air and bone to exclude sharp edges
Chun et al [41]	Structure sharpness/alteration of structure	Automatic	Abdomen	ROI	Using a structure coherence feature (SCF), homogeneous and structure edge regions were defined. Structure sharpness and alteration were evaluated using the SCF and SDs between homogeneous and structured regions

*ROI* region-of-interest, *HU* Hounsfield unit, *IQ* image quality, *ESF* edge spread function, *CTA* CT angiography, *ERS* edge rise slope, *MTF* modulation transfer function, *FWHM* full width half maximum, *TTF* task transfer function, *SCF* structure coherence feature, *SD* standard deviation

#### Manual methods

Manual methods for assessing spatial resolution predominantly focused on edge sharpness or slope measurements of a line profile across specific anatomical structures, i.e., edges in the head/brain region [29–33], in vascular structures such as coronary arteries and the portal vein [34–37] and in the intestines [38]. These methods typically involved drawing a single or multiple lines perpendicular to the edge of a structure to obtain a profile of the CT density values along the line, which shows a change in values at the edge. The spatial resolution or sharpness is subsequently calculated by determining the maximum slope of the line profile or differences between CT values at either side of the edge. These methods are effective for consistent comparisons of spatial resolution within defined regions and for specific protocols. However, they require radiological expertise for accurate line/ROI placement, making them time-intensive and prone to observer variability. Additionally, the localized nature limits the ability to represent global spatial resolution. Anatomical variability, such as differences in vessel diameter or tissue composition, can further affect results even with standardized scanning parameters. Partial volume effects and complex anatomical structures may also distort sharpness measurements, reducing the generalizability of these methods [30]. Additionally, to determine the maximal slope, Cao et al also considered the skewness of the CT number histogram in the liver parenchyma as a measure for image texture [37].

#### Automatic methods

Sanders et al [39] introduced the CT Resolution Index, which evaluates spatial resolution by measuring the edge spread function (ESF) across the air-skin interface after segmenting the patient’s skin and generating a tetrahedral mesh. While this method allows for quick, objective assessments across different anatomical regions, it is sensitive to noise and depends heavily on accurate segmentation. Jeukens et al [12] enhanced Sanders’ method by adding normalization and averaging steps but maintaining the dependence on accurate segmentation. Both methods offer the benefit of independence from specific body areas, as they can be consistently applied to the skin-air interface.

Other approaches, by Salimova et al [40] and Chun et al [41], focused on specific anatomical areas or tissue structures. Salimova’s method assessed image sharpness in the lower extremities by first segmenting and excluding air and bone edges, then analyzing the 2D image gradient of automatically detected significant edges per slice. Chun et al developed a structure coherence feature (SCF) technique to distinguish between homogeneous and edge-containing regions within the liver. In the edge-containing regions, the SCF is determined based on the presence of edges and the directionality of the edges. Similar to other automatic techniques, they remain constrained by dependencies on high-quality segmentation.

### Others

In addition to conventional metrics like noise, spatial resolution, and contrast, various other methods have been developed to objectively assess IQ in CT scans (Tables 4 and S4 for more details). These approaches often combined multiple image attributes and computational models to provide a more nuanced evaluation of IQ. The seven studies encompassed one manual method [42], four automatic methods [43–46] and two AI methods [10, 47].

**Table 4:** Objective CT image quality assessment methods not solely focusing on noise, contrast or spatial resolution

Study	Methodology	Technique type	Body region	ROI or Global	Key principle
Svalkist et al (2022) [42]	2D power spectrum (PS)	Manual	Chest	ROI	IQ was quantified in terms of the spatial frequency distribution with a 2D PS per slice, by averaging the PS of non-overlapping ROIs on each slice (ROIs were mirrored for artifact reduction)
Cheng et al [43]	Clarity	Automatic	Lesions	ROI	Combination of the modulation transfer function on the clinical CT images and the NPS calculated on previously made phantom images
Samei et al [44]	Task-dependent estimability index	Automatic	Stenosis quantification in CTA	ROI	Combination of motion point spread functions, CT image motion blur, noise and an automated maximum-likelihood estimator, to form an estimability index e’ as a task-based measure of IQ for stenosis quantification in cardiac CTA
Smith et al [45]	Detectability index	Automatic	Abdomen	Global	Combination of noise, spatial resolution and a reference task function to form a detectability index for the detection of lesions in the liver
Smith et al [46]	Detectability index	Automatic	Abdomen	ROI	Combination of image-derived noise, spatial resolution and a reference task function to calculate a detectability index for the detection of lesions in the liver
Lee et al [10]	Perceptual IQ	AI	Chest and abdomen	ROI	Self-supervised training strategy for object detection that results in a quantitative quality of CT images
Nakanishi et al [47]	General IQ	AI	CCTA	Global	Machine learning algorithm that predicts an IQ score, with features as noise, contrast, misregistration scores and uninterpretability index as input, meant to predict diagnostic / non-diagnostic

*ROI* region-of-interest, *PS* power spectrum, *IQ* image quality, *NPS* noise power spectrum, *CTA* CT angiography, *AI* artificial intelligence, *CCTA* coronary CT angiography

#### Manual methods

Svalkist et al analyzed the frequency components of clinical CT scans by computing the power spectrum (PS) from reconstructed image slices. By averaging the PS from non-overlapping pixel regions, mirroring to reduce edge artifacts and radially averaging the PS of each image slice, the study quantified the spatial frequency distribution [42].

#### Automatic methods

One significant aspect of IQ is how clearly a lesion or abnormality is rendered in a scan, for which Cheng et al introduced the term ‘clarity’ [43]. This metric combines the spatial resolution via the modulation transfer function (MTF) of the clinical image at hand with the noise texture derived from phantom images, matching the clinical scan protocol. It is assumed that the noise in the phantom images matches the clinical noise. However, its dependence on pre-acquired phantom images limits its applicability across different imaging settings.

Task-specific methods were a key focus among the identified automatic methods, valuable in contexts where the primary task is not to quantify the IQ in general, but how well the system can detect and characterize specific features required for a specific task within the image. Samei et al introduced a task-dependent estimability index for quantifying stenosis using cardiac CT angiography (CTA). This method integrated multiple objective factors, such as motion blur, noise, and spatial resolution, to quantify how accurately the imaging system can assess stenosis in cardiac vessels [44]. Similarly, Smith et al developed a detectability index with a model observer function for evaluating the system’s ability to detect liver lesions. This approach combined spatial resolution and noise measurements from previous validated techniques [18, 39] extracted from the clinical image with a reference task function (detecting a disk lesion with −15 HU contrast) to provide a metric for lesion detectability [45, 46]. While highly relevant for their respective clinical applications, these task-focused metrics are often limited in their generalizability to other anatomical regions or imaging tasks.

#### AI methods

Lee et al developed an AI-based approach that used a self-supervised training strategy to assess the perceptual IQ of chest and abdominal CT scans [10]. This method aimed to provide a quantitative score that reflects the overall quality of the image as perceived by radiologists, offering a more standardized and potentially more reliable measure of IQ. Nakanishi et al addressed the challenge of generalizability in AI methods by training a model for diagnostic versus non-diagnostic image classification of coronary CTA scans on multi-center and multi-vendor datasets. This approach increased the robustness of the AI model across various imaging systems [47].

## Discussion

A systematic review of the literature up to June 2024 was performed on objective diagnostic IQ assessment methods. An overview was provided by categorizing the methods in ‘noise,’ ‘contrast,’ ‘spatial resolution’ and ‘others’ and further dividing them into manual, automatic and AI-based methods. Thirty-five studies were identified that proposed or employed reference-free IQ methods beyond traditional methods. The traditional methods encompassed IQ assessment performed within a manually selected ROI by determining the SD of the HU values, which can be used to determine the SNR or CNR. As these methods are very well-known but limited in their ability to capture more complex aspects of IQ, this review focuses on more advanced IQ assessment methods.

There is an ongoing transition towards more advanced, automated and AI-driven IQ assessment techniques, which offer opportunities for large-scale, real-time, and reproducible assessments. ROI-based methods are still regarded as valuable for localized and task-specific evaluation. However, they are time-consuming and less reproducible and inherently limited in generalizability due to observer dependence, leading to variability in shape (square, circular, line) and size. The American Association of Physicists in Medicine recommends an ROI size of at least 1 cm in diameter for reliable measurements [24, 48]. Large ROI sizes are not always practical in clinical settings, especially when dealing with small structures, in images that have a complex anatomy or in regions with limited homogeneous tissue. These manual placement challenges underscore the need for automatic and AI-based methods, offering advantages in standardization and scalability. This would eliminate the need for user input, improve reproducibility, and align with the goal to achieve real-time integration of IQ assessment into scanner workflows and quality control programs.

AI-based approaches, particularly CNNs, are promising for IQ assessment. When trained on subjectively assessed datasets, CNNs can simulate subjective IQ assessment while providing greater consistency. These models have demonstrated accuracy in automating noise and general IQ measurements, trained on anthropomorphic phantom data and validated with clinical datasets [10, 24, 25, 47]. However, AI methods face challenges, such as the need for large, diverse, annotated datasets, their limited generalizability across different body regions and examination protocols. They are difficult to validate due to the lack of a clinical universal IQ “gold standard.” Additionally, many automated and AI-based approaches also rely on accurate image segmentation, which are often prone to failure due to unexpected anatomy or artifacts.

Two noticeable gaps in the existing literature were identified: First, a lack of comprehensive metrics that integrate multiple IQ characteristics (e.g., noise, contrast, spatial resolution) into a single IQ score. Second, absence of robust, objective validation frameworks of IQ assessment methods, as there is no reference or “gold standard” to compare results.

Looking forward, CT IQ assessment should move toward a fully objective approach to overcome human variability. Rather than evaluating isolated aspects of IQ, IQ assessment should comprise a single IQ score that accounts for all relevant parameters within the context of the intended diagnostic task. In parallel, validation frameworks should shift toward objective, task-based reference standards to replace commonly used subjective assessments. Relying on subjective methods as the ultimate truth and “gold standard” to validate new objective approaches can introduce circular reasoning, particularly when the new methods aim to surpass the limitations of subjective ones. Consequently, claims of accuracy or robustness of the IQ assessment methods may be overstated. Ideally, IQ assessment should be embedded into the scanner’s acquisition or reconstruction pipeline, offering real-time feedback. This would support proactive quality assurance, can help with scan and reconstruction protocol optimization, and even lead to a reduction of unnecessary repeat scans.

As researchers are constantly striving to optimize CT IQ for each individual patient and examination [2], and in particular to balance IQ with radiation dose, it would be beneficial to keep improving existing IQ evaluation methods. Future research should prioritize the development of automated methods to prevent observer variability due to manual intervention. There should be a focus on robust segmentation-free approaches or improvements in segmentation reliability. Additionally, expanding training datasets for AI models, including diverse imaging conditions, patient demographics, and scanner technologies, will largely improve the potential of AI in IQ assessment, but this requires collaboration and data sharing between institutions. Finally, this review highlights the need for standardized metrics and benchmarks in objective IQ assessment, as well as the need for reliable validation methods. Establishing common frameworks would improve the reproducibility of IQ research and further foster the development of clinically meaningful IQ evaluation methods.

## Conclusion

By giving an overview of existing reference-free advanced objective IQ assessment methods, this review shows the progression from manual ROI-based techniques to fully automated and/or AI-based approaches. Further development of objective IQ assessment methods is required, including methods integrating multiple IQ parameters, expanding training datasets for AI models, and developing robust segmentation-free methods. While no single method can yet be considered universally optimal, this review provides a structured overview to guide in selecting currently existing IQ assessment methods appropriate to their specific goals.

## Supplementary information

ELECTRONIC SUPPLEMENTARY MATERIAL

## Abbreviations

AI: Artificial intelligence
CNN: Convolutional neural network
CNR: Contrast-to-noise ratio
CTA: CT angiography
ERS: Edge rise slope
ESF: Edge spread function
FWHM: Full width half maximum
GNI: Global noise index
GNL: Global noise level
HU: Hounsfield unit
IQ: Image quality
NPS: Noise power spectrum
PS: Power spectrum
ROI: Region-of-interest
SCF: Structure coherence feature
SD: Standard deviation
SNR: Signal-to-noise ratio
TTF: Task transfer function
