# Supplementary material for: Objective assessment of diagnostic image quality in CT scans: what radiologists and researchers need to know
Source: Insights Imaging. 2025 Jul 10;16:154. doi: 10.1186/s13244-025-02037-y (PMC12246289; doi:10.1186/s13244-025-02037-y)
Supplement: Supplementary file 1 — ELECTRONIC SUPPLEMENTARY MATERIAL [file 13244_2025_2037_MOESM1_ESM.docx]

**Supplementary Material**

This supplementary material contains more detailed information on the 35 studies included in the review. Tables S1 to S4 complement Tables 1 to 4 by providing additional information on the key advantages and disadvantages as well as method of validation.

| **TABLE S1 –** Objective CT image quality assessment methods focusing on noise  *ROI, region-of-interest; NPS, noise power spectrum; PCD-CT, photon-counting detector CT; SD, standard deviation; GNL, global noise level, GNI, global noise index, AI, artificial intelligence;* | | | | | | | | |
| --- | --- | --- | --- | --- | --- | --- | --- | --- |
| **Study** | **Methodology** | **Technique type** | **Body region** | **Key principle** | **ROI or**  **Global** | **Key advantages /**  **disadvantages** | **Method of validation** | |
| Zhang et al. (2024) | NPS | Manual | All | Novel mathematical method that uses the raw counts data of PCD-CT scan to obtain a local NPS | ROI | (+) Enables NPS measurement without exposing a patient to repeated radiation  (-) Only applicable to PCD-CT scans  (-) Depends on correct placement of ROI by expert | Validated with a conventional local NPS measurement method using repeated scans on phantom data | |
| Smith et al. (2021) | NPS | Automatic | Abdomen | Segmenting the liver, selecting a uniform area within it and measuring noise patterns within the data via noise auto covariance | Global | (+) Also measurement over complete CT-series is described  (-) Method cannot fully rule out possible anatomical “contamination” | Validated with anthropomorphic phantom data with ground-truth NPS measurements | |
| Kortesniemi et al. (2008) | Edge-preserving mask-filtering algorithm | Automatic | All | Creating a map of filtered local minimum SD’s using a moving square mask, forming an IQ score | Global | (+) Works on slices but method can easily be extended to complete CT-series  (+) Relatively independent of high-detail content  (+) Not anatomy dependent | Validated with technical and anthropomorphic phantom data with noise measured in manually placed ROIs in the background and by testing the method on a clinical head CT scan  Also validated in Jeukens et al. (2023) and Franck et al. (2017) | |
| Christianson et al. (2015) | GNL | Automatic | Abdomen | Finding the most frequent noise level in areas of homogeneous tissue | Global | (-) Small-scale anatomic texture may contribute to the global noise level  (-) Depends on successful threshold based segmentation  (-) Requires sufficiently large region of soft tissue | Validated with phantom data with noise measured by subtracting repeated scans and validated with an observer study via the SD in manually placed ROIs on clinical abdominal CT scans of 6 patients | |
| Anam et al. (2021) | GNL | Automatic | All | Finding minimum standard deviation in any region of the body as a measure for noise | Global | (+) Applicable to all body regions  (+) Not dependent on segmentation  (-) Only validated on abdominal data | Validated with results of noise measurements from Christianson et al. (2015) on a homogeneous phantom and tested on clinical abdominal CT scans of 27 patients | |
| Alsaihati et al. (2023) | GNI | Automatic | Soft tissue | Enhanced version of Christianson et al. (2015) but averaging it over slices to obtain the GNI of a complete series | Global | (+) Gives noise of a complete CT-series  (-) Depends on successful segmentation  (-) Requires large region of soft tissue  (-) Small-scale anatomic texture may contribute to the global noise level | Compared to results of Christianson et al. (2015) | |
| Tian et al. (2016) | Subtraction technique | Automatic | Abdomen, lung | Subtracting sequential slices and filtering out edges to measure SD in resultant uniform area | Global | (+) GNI cannot be influenced by anatomical variability  (-) Lower prediction accuracy on lung scans  (-) Requires large enough uniform areas | Validated with true noise map (subtraction of consecutive scans) of phantom data (turkey) and with the SD in manually placed ROIS on clinical chest and abdominal CT scans of 83 patients | |
| Malkus et al. (2017) | GNI | Automatic | All | Determination of the mode/mean of the SD in air, by subtracting adjacent slices and with an edge-finding algorithm to remove morphological variations | Global | (-) Sufficient amount of air is needed around the patient | Validated with results of the noise measurement method from Tian and Samei et al. (2016) measured inside a phantom and with the SD in manually placed ROIs on clinical abdominal CT scans of 40 patients | |
| Jeukens et al. (2023) | GNL | Automatic | All | Combines Malkus et al. and Christianson et al. by measuring GNL in soft tissue and air | Global | (+) GNL in air cannot be influenced by anatomical variability  (+) GNL in air is not limited to body region.  (-) Depends on successful threshold based segmentation | Validated with an observer study on anthropomorphic phantom data and clinical abdominal CT scans of 30 patients via subjective IQ assessment (overall diagnostic IQ) | |
| Chun et al. (2015) | Automated noise measurement with a novel structure coherence feature | Automatic | Regions with fat | Calculation of the structure coherence feature to determine homogeneous ROIs and using the average SD as noise level | ROI | (+) Small anatomical structures do not influence the noise results  (+) Not limited to body region except for head (not enough fat)  (-) Depends on successful segmentation | Validated with the SD in manually placed ROIs on clinical chest and abdominal CT scans of 100 patients | |
| Ketola et al. (2024) | Deep learning-based image noise quantification framework | AI | Chest | Convolutional neural network trained on anthropomorphic phantom data labeled with true noise maps via 2 consecutive scans | Global | (-) AI is only trained on phantom data and generalizability to human anatomy is dependent on the ability to include all relevant features in the phantom training data | Validated with true noise maps of phantom data and with noise maps (subtracting 5-slice running average) of clinical chest CT scans of 5 patients | |
| Huber et al. (2023) | Deep learning-based image noise quantification framework | AI | Chest, head and pelvis | Convolutional neural network trained on anthropomorphic phantom data labeled with true noise maps with per-pixel SDs from 100 replicate scans | Global | (-) AI is only trained on phantom data and generalizability to human anatomy is dependent on the ability to include all relevant features in the phantom training data | Validated with true noise maps of phantom data and with SD in manually placed ROIS on clinical CT scans of 10 patients | |
|  | | | | | | | |  |

| **TABLE S2** – Objective CT image quality assessment methods focusing on contrast  *ROI, region-of-interest; HU, Hounsfield Unit; CNR, contrast-to-noise ratio; SNR, signal-to-noise ratio; IQ, image quality; CTA, computed tomography angiography;* | | | | | | | |  |
| --- | --- | --- | --- | --- | --- | --- | --- | --- |
| **Study** | **Methodology** | **Technique type** | **Body region** | **Key principle** | **ROI or Global** | **Key advantages / disadvantages** | **Method of validation** | |
| Beer et al. (2019) | Line-density profile analysis | Manual | Abdomen | Difference between maximum and minimum HU values on the line | ROI | (-) Local contrast assessment, not completely representative for full image  (-) Depends on correct placement of the line profile by expert | Compared to CNR/SNR measurements and to results of subjective IQ (vessel contrast) assessment via a Likert score on clinical abdominal CT scans of 45 patients | |
| Pallenberg et al. (2020) | Automated mean HU measurement of aortic CTA volumes | Automatic | Aorta | Classification of insufficient, optimal or excessive contrast based on the mean HU values in ROIs that were automatically positioned in the CTA volume (CTA image slices were automatically localized as well) | ROI | (-) Automatic ROI placement shows varying results  (-) Slice detection works with a template matching approach which requires sufficient reference templates | Validated with manual contrast measurements on clinical CTA scans of 73 patients | |
| Abadi et al. (2017) | Automated HU distribution measurement | Automatic | Chest (lung, liver, aorta, spine) | Median of the distribution of HUs by segmentation of organs and automated ROI placement | ROI | (+) Shown to work on both contrast and non-contrast-enhanced exams  (-) Depends on successful segmentation | Validated with manual contrast measurements on clinical chest CT scans of 732 patients | |
| Jeukens et al. (2023) | Automated HU distribution measurement | Automatic | Abdomen | Generating an histogram of HUs after removing air and bone with threshold based segmentation. Various contrast metrics are defined with the histogram analysis | Global | (+) Works on entire scan area instead of specific ROI locations  (-) Shape of histogram is dependent on contrast protocol and anatomical area | Validated with an observer study on anthropomorphic phantom data and clinical abdominal CT scans of 30 patients via subjective IQ assessment (overall diagnostic IQ) | |
|  | | | | | | | |  |

| **TABLE S3** – Objective CT image quality assessment methods focusing on spatial resolution  *ROI, region-of-interest; HU, Hounsfield Unit; IQ, image quality; ESF, edge spread function; CTA, CT-angiography; ERS, edge rise slope; MTF, modulation transfer function; FWHM, full width half maximum; TTF, task transfer function; SCF, structure coherence feature; SD, standard deviation* | | | | | | | |  |  |
| --- | --- | --- | --- | --- | --- | --- | --- | --- | --- |
| **Study** | **Methodology** | **Technique type** | **Body region** | **Key principle** | **ROI or Global** | **Key advantages / disadvantages** | **Method of validation** | | |
| Korn et al. (2013) | Sharpness | Manual | Head | Sharpness was quantified in terms of the maximal slope (change in HU per pixel) across a line profile perpendicular to the skull circumference | ROI | (+) Practical for easy comparison of spatial resolution between CT-scans made with the same protocol  (-) Requires expert in radiology for manual ROI drawing  (-) Local spatial resolution assessment, not completely representative for full image | Compared to results of subjective IQ (sharpness) assessment via a Likert score on clinical head CT scans of 15 patients | | |
| Brodoefel et al. (2014) | Sharpness | Manual | Head | Sharpness was quantified in terms of the gradient or maximal slope (change in HU per pixel) across a line profile perpendicular to the skull circumference | ROI | (+) Practical for easy comparison of spatial resolution between CT-scans made with the same protocol  (-) Requires expert in radiology for manual ROI drawing  (-) Local spatial resolution assessment, not completely representative for full image | Compared to results of subjective IQ (sharpness) assessment via a Likert score on clinical head CT scans of 25 patients | | |
| Ernst et al. (2016) | Sharpness | Manual | Head | Sharpness was quantified in terms of the maximal slope (change in HU per pixel) across a line profile perpendicular to the skull circumference | ROI | (+) Practical for easy comparison of spatial resolution between CT-scans made with the same protocol  (-) Requires expert in radiology for manual ROI drawing  (-) Local spatial resolution assessment, not completely representative for full image | Compared to results of subjective IQ (sharpness) assessment via a Likert score on clinical head CT scans of 24 patients | | |
| Wu et al. (2020) | Edge spread function | Manual | Head | Spatial resolution was quantified in terms of the width of the edge spread function (ESF) across line profiles perpendicular to the boundary between the lateral ventricle and surrounding brain parenchyma | ROI | (+) Practical for easy comparison of spatial resolution between CT-scans made with the same protocol  (-) Requires expert in radiology for manual ROI drawing  (-) Local spatial resolution assessment, not completely representative for full image | Observer study is subject of ongoing work | | |
| Altmann et al. (2023) | Sharpness | Manual | Head/Neck | Sharpness was quantified in terms of the maximum slope (change in HU per pixel) across line profiles perpendicular to a border between fat and muscle tissue | ROI | (+) Practical for easy comparison of spatial resolution between CT-scans made with the same protocol  (-) Requires expert in radiology for manual ROI drawing  (-) Local spatial resolution assessment, not completely representative for full image | Compared to results of subjective IQ –(sharpness) assessment via a Likert score on clinical head and neck CT scans of 40 patients | | |
| Mergen et al. (2022) | Sharpness | Manual | Coronary arteries | Sharpness was quantified in terms of the maximum slope (change in HU per pixel) across line profiles perpendicular to the left anterior descending artery­­­ | ROI | (+) Practical for easy comparison of spatial resolution between CT-scans made with the same protocol  (-) Requires expert in radiology for manual ROI drawing  (-) Local spatial resolution assessment, not completely representative for full image | Compared to results of subjective IQ (vessel and plaque delineation) assessment via a Likert score on clinical CTA scans of 20 patients | | |
| Feger et al. (2015) | Sharpness | Manual | Coronary arteries | Sharpness was quantified in terms of the difference between 25% and 75% of the maximal HU value and in terms of the maximal slope (change in HU per pixel) across a line profile perpendicular to the edge of a vessel | ROI | (+) Practical for easy comparison of spatial resolution between CT-scans made with the same protocol  (-) Requires expert in radiology for manual ROI drawing  (-) Local spatial resolution assessment, not completely representative for full image | Not compared to or validated with other IQ methods | | |
| Heinrich et al. (2023) | Sharpness | Manual | Coronary arteries | Sharpness was quantified in terms of the maximum slope (change in HU per pixel) across a line profile perpendicular to the edge of the vessel lumen | ROI | (+) Practical for easy comparison of spatial resolution between CT-scans made with the same protocol  (-) No direct correlation was visible with subjective assessment  (-) Requires expert in radiology for manual ROI drawing  (-) Local spatial resolution assessment, not completely representative for full image | Compared to results of subjective IQ (how well images were suited for performing specific tasks) assessment via a Likert score on clinical CTA scans of 50 patients, but no direct correlation was visible | | |
| Cao et al. (2023) | Edge rise slope (ERS)  Image texture | Manual  Manual | Abdomen  Abdomen | Sharpness was quantified in terms of the ERS (the ratio between the difference between the first peak and last dip in HU values, divided by their distance) across a line profile perpendicular to the edge of a portal vein  Skewness of the CT number histogram on the relatively uniform area of the liver parenchyma | ROI  ROI | (+) Practical for easy comparison of spatial resolution between CT-scans made with the same protocol  (-) Requires expert in radiology for manual ROI drawing  (-) Local spatial resolution assessment, not completely representative for full image | Compared to results of subjective IQ (sharpness) assessment via a Likert score on clinical head CT scans of 40 patients | | |
| Johnson et al. (2014) | Sharpness | Manual | Intestines | Sharpness was quantified in terms of the edge-width across an ROI crossing the psoas-fat edge | ROI | (+) Practical for easy comparison of spatial resolution between CT-scans made with the same protocol  (-) Requires expert in radiology for manual ROI drawing  (-) Local spatial resolution assessment, not completely representative for full image | Validated by testing the method on clinical data of 5 patients with added blur | | |
| Sanders et al. (2016) | CT Resolution index based on the edge spread function | Automatic | Skin | Spatial resolution was quantified by segmentation of the patient’s body and measuring the edge spread function (ESF) across line profiles perpendicular to the air-skin interface | Global | (+) Independent of scanned body area (-) Higher noise will make it harder to extract clean ESFs  (-) Highly dependent on successful segmentation | Validated with MTF measurements on phantom data and compared to results of subjective IQ (sharpness) assessment via a two-alternative-forced-choice methodology on clinical abdominal and chest CT scans of 72 pairs of edges extracted from different images | | |
| Jeukens et al. (2023) | FWHM of the Task transfer function (TTF) | Automatic | Skin | Enhanced version of the method of Sanders et al. (2016) by calculating the FWHM from the ESF | Global | (+) Independent of scanned body area  (-) Highly dependent on successful segmentation | Validated with an observer study on anthropomorphic phantom data and clinical abdominal CT scans of 30 patients via subjective IQ assessment (overall diagnostic IQ) | | |
| Salimova et al. (2022) | Image sharpness | Automatic | Lower extremities | Sharpness was quantified in terms of the 2D image gradient on a per-slice bases after automated segmentation of air and bone to exclude sharp edges | Global | (-) Depends on successful segmentation of bone structures | Compared to results of subjective IQ assessment (overall diagnostic IQ) via a Likert score on clinical CTA scans of 100 patients | | |
| Chun et al. (2022) | Structure sharpness/ Alteration of structure | Automatic | Abdomen | Using a structure coherence feature (SCF) homogeneous and structure edge regions were defined. Structure sharpness and alteration were evaluated using the SCF and SD’s between homogeneous and structured regions | ROI | (+) Method can differentiate between homogeneous area and structural transitions  (+) Tested on scans from different vendors  (-) Depends on successful liver segmentation via deep learning segmentation model | Validated with manually measured edge slopes on clinical abdominal CT scans of 120 patients and good agreement is found | | |
|  | | | | | | | | |  |

| **TABLE S4** – Objective CT image quality assessment methods not solely focusing on noise, contrast or spatial resolution  *ROI, region-of-interst; PS, power spectrum; IQ, image quality; NPS, noise power spectrum; CTA, CT angiography; AI, artificial intelligence; CCTA, coronary CT angiography;* | | | | | | | | |  |
| --- | --- | --- | --- | --- | --- | --- | --- | --- | --- |
| **Study** | **Methodology** | **Technique type** | **Body region** | **Key principle** | **ROI or Global** | **Key advantages / disadvantages** | **Method of validation** | | |
| Svalkist et al. (2022) | 2D power spectrum (PS) | Manual | Chest | IQ was quantified in terms of the spatial frequency distribution with a 2D PS per slice, by averaging the PS of non-overlapping ROIs on each slice (ROIs were mirrored for artefact reduction) | ROI | (+) Can be extended to 3D to be applicable to a complete series  (+) Captures both noise and spatial resolution characteristics  (-) No clear correlation was observed with subjectively assessed IQ | Compared to subjective IQ (overall diagnostic IQ) assessment via a Likert score on clinical chest CT scans of 25 patients | | |
| Cheng et al. (2019) | Clarity | Automatic | Lesions | Combinination of the modulation transfer function on the clinical CT images and the NPS calculated on previously made phantom images | ROI | (-) Requires the NPS of previously made phantom CT-images with the same scanning protocol, potentially hampering generalizability | Validated with an observer study based on ranking 23 sets of up to 6 clinical abdominal CT scans with respect to their IQ | | |
| Samei et al. (2021) | Task-dependent estimabilility index | Automatic | Stenosis quantification in CTA | Combination of motion point spread functions, CT image motion blur, noise and an automated maximum-likelihood estimator, to form an estimability index e’ as a task-based measure of IQ for stenosis quantification in cardiac CTA | ROI | (+) Framework accounts for a wide range of influential factors, including cardiac motion, spatial resolution, noise and others  (-) Framework is only usable to quantify stenosis estimation task performance, not generalizable | Validated with subjective IQ assessment using a 4-point Likert scale on clinical CTA scans of 132 patients | | |
| Smith et al. (2018) | Detectability index | Automatic | Abdomen | Combination of noise, spatial resolution and a reference task function to form an detectability index for the detection of lesions in the liver | Global | (+) Method is not limited to one specific reconstruction algorithm but can compare images made with different reconstruction algorithms  (-) IQ parameter is specifically designed for lesion detection in liver scans and not easily generalizable  (-) Method relies on phantom images made at forehand | Validated with an observer detection study on clinical abdominal CT scans of 21 patients | | |
| Smith et al. (2021) | Detectability index | Automatic | Abdomen | Combination of image derived noise, spatial resolution and a reference task function to calculate a detectability index for the detection of lesions in the liver | ROI | (+) This method does not need phantom scans, as opposed to Smith et al. (2018). | Validated on clinical abdomen scans against the detection accuracy in an observer study of clinical abdominal CT scans of 21 patients | | |
| Lee et al. (2022) | Perceptual IQ | AI | Chest and abdomen | Self-supervised training strategy for object detection that results in a quantitative quality of CT images | ROI | (+) Study shows potential generalizability to other anatomies | Tested on clinical and anthropomorphic phantom CT images among different dose levels and validated with subjective IQ assessment via a Likert score on clinical CT scans of 30 patients | | |
| Nakanishi et al. (2018) | General IQ | AI | CCTA | Machine learning algorithm that predicts an IQ score, with features as noise, contrast, misregistration scores and un-interpretability index as input, meant to predict diagnostic / non-diagnostic | Global | (+) Trained on multicenter data (different CT-scanners)  (-) Algorithm might be biased because training data contained more good than poor IQ | Validated with subjective IQ assessment via a Likert score on clinical CCTA studies of 172 patients | | |
|  | | | | | | | |  |  |
